# Locust Bean Gum, a Vegetable Hydrocolloid with Industrial and Biopharmaceutical Applications

**DOI:** 10.3390/molecules27238265

**Published:** 2022-11-26

**Authors:** Max Petitjean, José Ramón Isasi

**Affiliations:** Department of Chemistry, University of Navarra, 31080 Pamplona, Spain

**Keywords:** locust bean gum, galactomannans, carob, thickening agents

## Abstract

Locust bean gum (LBG), a vegetable galactomannan extracted from carob tree seeds, is extensively used in the food industry as a thickening agent (E410). Its molecular conformation in aqueous solutions determines its solubility and rheological performance. LBG is an interesting polysaccharide also because of its synergistic behavior with other biopolymers (xanthan gum, carrageenan, etc.). In addition, this hydrocolloid is easily modified by derivatization or crosslinking. These LBG-related products, besides their applications in the food industry, can be used as encapsulation and drug delivery devices, packaging materials, batteries, and catalyst supports, among other biopharmaceutical and industrial uses. As the new derivatized or crosslinked polymers based on LBG are mainly biodegradable and non-toxic, the use of this polysaccharide (by itself or combined with other biopolymers) will contribute to generating greener products, considering the origin of raw materials used, the modification procedures selected and the final destination of the products.

## 1. Introduction

Locust bean gum (LBG) is a high molecular weight non-ionic galactomannan polysaccharide, extracted from the seeds of *Ceratonia Siliqua* (carob tree, or locust bean tree, mainly found in the Mediterranean region). Although both structurally and chemically similar to guar gum, it shows important differences. Soluble in water with the addition of heat, LBG solutions do not form gels by themselves but enhance those produced by other types of hydrocolloids such as xanthan and carrageenan.

Among the many constituents of the carob fruit, including sugars and bioactive compounds [1,2,3,4,5], their polysaccharides are in both the carob fiber and the carob bean gum [6,7]. Recently, potential health benefits of carob products have been reported because the polyphenols in the fruit are powerful antioxidants [2,3], and the dietary fibers and sugars prevent diabetes, heart diseases and gastrointestinal disturbances [6]. The carob mucilage, also known as locust bean gum, was critically described at the end of the 19th century, and its colloidal properties were well-studied many years ago [8].

As mentioned by Gioxary et al. in their recent review, the development of carob tree cultivation can be useful for the environment thanks to the capacity of this species to prevent soil degradation, besides its high CO_2_ absorption ratio and its potential usage not only to produce animal feed but also for the human diet in the Mediterranean regions [1].

LBG is mainly used as an additive (E410) in the food and beverage industry, most often as a thickening, stabilizing and gelling agent, or emulsifier, the texture being an intangible property of food of great importance [9]. This polysaccharide has found applications in other sectors such as pharmaceuticals, cosmetics, textiles, paper, or the petroleum industry [10,11,12]. In fact, various non-starch polysaccharides isolated from plants, including LBG, show a considerable potential to prepare drug delivery systems to achieve tailored and/or site-specific drug release [10]. In addition, natural gum-based hydrogels can be used in tissue engineering, wound dressing, hygienic products, agriculture, and water purification [11].

In this review, after the description of the structural characteristics of locust bean gum, its biosynthetic origin and its chemical isolation will be also accounted. Its chemical composition originates a particular conformation in aqueous solutions, responsible for its rheological properties, also presented here. Besides the applications of these solutions due to their own viscosity, an essential aspect is their synergistic behavior when mixed with other polysaccharides. The last section of this review deals with the derivatives of locust bean gum, either by functionalization or cross-linking.

## 2. Structure, Processing and Properties

### 2.1. Composition

**Figure 1:**
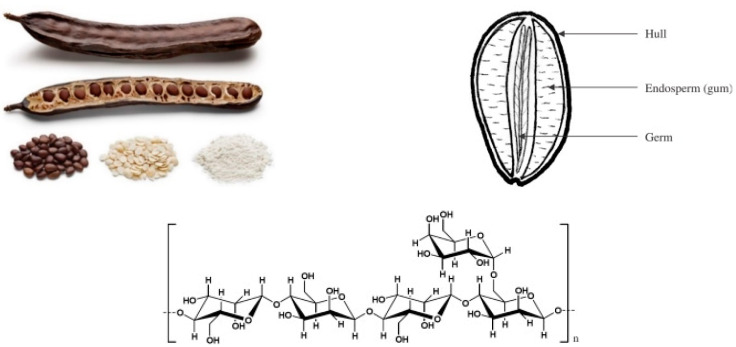
Carob fruit and locust bean gum.

Locust bean gum (LBG), a polysaccharide of vegetal origin that belongs to the galactomannan family [13], is composed of β-(1-4)-mannose backbones randomly branched by α-(1-6)-galactose (Figure 1) [14]. The mannose:galactose ratios, usually determined by the Blakeney method [15], are different for each type of galactomannan, depending on their origin [16]. Thus, the ratios found are between 1 to 1 and 10 to 1 [14,17] for different gums: fenugreek gum 1:1; guar gum 2:1; tara gum 3:1; LBG 4:1; cassia gum 5:1, etc. [18]. The first elucidation of the fine structure of LBG was proposed by Baker et al. in 1975, using an alkaline degradation method [19], but its discovery and analysis come from much earlier, as explained by Dea et al. [14]. There are other characterization techniques available, such as those involving hydrolysis, periodate oxidation [20], 13C NMR [21], methylation [22,23], partial or enzymatic hydrolysis [17] and the development of sulfonyl derivates [24].

LBG and other galactomannans can be obtained from the Leguminosae plant family. The carob tree, a Mediterranean plant also known as *Ceratonia siliqua*, can be found in Portugal, Spain, Italy, Cyprus, Greece, Morocco, and the rest of northern Africa, but it is also grown in Asia, Australia and South America. A pod of the carob bean will be biologically composed of a seed coat (≈30%), germ (≈25%) and endosperm (≈42%). The rest of the mass will be moisture (≈8%) [25]. Chemically, a pod is a mixture of galactomannan (≈85%), water (≈8%), protein (≈5%), ash, fibers and fat, each one around 1% [26].

### 2.2. Biosynthesis

The biosynthesis path takes place in the lumen of the Golgi apparatus. The product is then transported to the surface cell by secretory vesicles and introduced into its wall matrix [27]. The biosynthesis of galactomannan has been well described by Sharma et al. [28]. Briefly, it begins with the transformation of sucrose in uridine diphosphate (UDP)-glucose and UDP-fructose by a synthase, and in glucose and fructose by an invertase. Fructose is then phosphorylated and isomerized to produce mannose-6-phosphate. The phosphate group is delocalized into position 1 by phosphomannomutase. This mannose-1-phosphate is then transformed into GDP-mannose using GDP-mannose pyrophosphorylase. UDP-glucose, previously formed, is then converted into UDP-galactose by UDP-galactose 4-epimerase. The synthesis of both substrates, GDP-mannose and UDP-galactose, is also an enzymatic process. The mannan backbone is formed by using the GDP-mannose thanks to the mannan synthase, and the branched galactosyl units on the backbone come from UDP-galactose by the galactosyltransferase enzyme [29,30]. The M:G ratio can be modified in vitro by changing the GDP-mannose concentration [31] along the synthesis process, or by removing galactosyl units during hydrolysis thanks to α-galactosidase [32]. It is also modified by the culture conditions and depends on the seed origin and the gum fabrication process [14,33,34].

### 2.3. Extraction

The extraction of LBG can proceed following different methods. First, the removal of the seeds from the pod must be performed mechanically. After that, to eliminate the hull, diverse procedures are available, such as roasting [35], acid extraction [36,37], water extraction [36], mechanical processes, or by swelling and freezing [12]. The endosperm is then milled and pulverized under different conditions to remove the remaining husk. The endosperm is a mixture of polysaccharides, proteins, and other impurities, which necessitates a purification step, and this can be performed by precipitation coupled with dialysis. After dissolving the powder in water, the addition of an alcohol such as ethanol [38,39], methanol [40], or isopropanol [14]; a copper complex [41] or a barium-complex [42] produces the precipitation of the galactomannan [28]. Azero et al. studied different purification techniques and their impact on the physicochemical properties of the formed gum, and they showed better inter- and intramolecular associations for LBG for the one filtered over the centrifuged product [43]. Isopropanol decreases the content of ashes and proteins and produces a more stable solution due to the elimination of enzymes and impurities [33]. Dakia et al. compared two types of processes: the first one using water, removing the different seed layers by letting the seed swell in boiling water, and the germ removed after drying the seed; the second one by an acidic extraction. The seed is macerated in H_2_SO_4_/H_2_O 60/40 (*v*/*v*) at 60 °C for 1 h. The carbonized hull is removed by washing for 2 min with a metallic sieve. After drying the seeds, they are crushed to release and remove the germ. Both procedures mill and sift the endosperm using the same conditions [36]. Some physicochemical differences arise between the LBGs coming from the two processes: acid extraction produces better thickening properties, while water extraction is responsible for a higher solubility at high temperatures, for example.

### 2.4. Conformation

X-ray analysis shows that the LBG powder is mainly amorphous [21]. As shown by Grimaud et al., ordered conformations similar to that of the LBG backbone (i.e., a chain of α-(1-4)-mannose) favor the presence of crystalline structures and then an inter- or intramolecular complexation, which creates hydrophobic regions preventing good solubilization in water [44]. The galactose-branched units facilitate the solubilization of the backbone, and this property increases with the degree of substitution [45]. The conformation of the galactomannan depends on the inter- and intra-molecular interactions and on their hydrophobic interactions [46], passing from elongated ribbon-like forms [12] to aggregates, and, therefore, forming hydrophobic microdomains. These hydrophobic microdomains also depend on the distribution of galactose, the mannose:galactose ratio, and the solution temperature [47]. These characteristics also influence its critical association concentration. Molecular modeling showed the influence of the galactose-branched chains on the flexibility of the mannose backbone [48]. Then, a method to measure this flexibility by using the persistent length was found, which produces, for a 1:1 M:G, an error of 3 Å [45]. Other techniques have facilitated the study of the fine structure of galactomannans, such as X-ray scattering [49], one- or two-dimensional NMR [50], size exclusion chromatography coupled with multi-angle static light scattering [51] and fluorescence spectroscopy [47].

### 2.5. Physico-Chemical Properties

**Figure 2:**
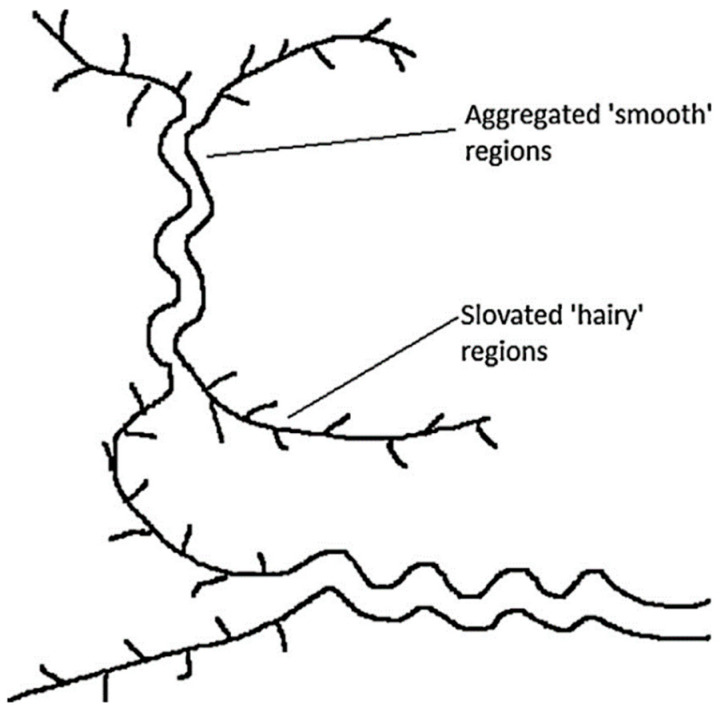
Entanglement of galactomannan polymers; the smooth regions form the insoluble part of the gel while the hairy regions permit the solubility of the polysaccharide [12].

The galactomannan aqueous solubility depends on the temperature but it is also associated with the M:G ratio. Thus, the higher the amount of branched galactose units present in the polysaccharide, the higher its solubility at a low temperature [52,53]. In that sense, for LBG, the solubility value originates from a thermodynamic equilibrium between the amorphous solid phase swollen by the solvent and the pure solvent phase [54]. Because LBG does not possess ionizable functions, solubilization depends on the amount of hydrogen bonds and the quality of the solvent [55]. As mentioned above, a pure mannose backbone possesses a high level of intra- and intermolecular interactions via hydrogen bonding, permitting the aggregation that leads to precipitation [44,56,57]. The galactose branched units have two functions: solubilization and anti-aggregation of the polysaccharide. The solubility temperature depends also on the distribution of galactosyl-branched units along the backbone (Figure 2). A high-temperature solubility means compact galactosyl branched units, so large smooth regions [58].

For dilute galactomannan solutions, the viscosity, as the solubility, will depend on the molecular mass, the M:G ratio and the distribution of the galactosyl branched units, as expressed by the Mark–Houwink equation [59]. A higher molar mass yields a higher intrinsic viscosity, as occurs for a large number of galactosyl-branched units [14,60]. Therefore, Morris et al. proposed an equation introducing the gyration radius related to the distribution of galactosyl-branched units [61]. For a higher concentration, the polymer interpenetration phenomenon increases the viscosity by creating a physical covering [62]. If this phenomenon is the only one taking place in the solution, the theoretical viscosity will depend merely on the concentration and molar mass [63]. However, LBG solutions have a higher viscosity than the theoretical ones, meaning that another phenomenon is also occurring. This, as explained by Sittikijyothin et al., consists of the ‘hyperentanglements’ [64], an intermolecular aggregation influenced by the M:G ratio and galactose distribution [65]. A higher amount of smooth regions or a lower quantity of galactose branched units permits the formation of these additional entanglements [56,66,67].

The dynamic viscosity of galactomannans and, more specifically, that of LBG, has been well studied. As for many polysaccharides, this hydrocolloid shows a pseudoplastic behavior in solution [64,68]. The galactomannan concentration and its microstructure are two factors influencing the dynamic viscosity of the solutions; both of these characteristics are directly related to the intra- and intermolecular associations and, consequently, depend on the smooth locust bean gum region [66,69]. The ionic strength, temperature and pH have a small influence on the viscosity [14,17]. Temperature and pH can break the polysaccharides, modifying the final viscosity due to the changes in the molecular mass of the polymer chains. The solution temperature has an impact on its viscosity since a higher temperature permits an efficient solubilization process and also promotes a higher solubility [70]. When this solution is cooling down, the observed viscosity will be higher than that of the one that was first dissolved at a lower temperature. This can be explained by a higher entanglement probability when we have produced better solubilization of the hydrocolloid [60].

Galactomannans are mainly incorporated as a powder, solubilized in the desired solution and used, due to their rheological properties, for food improvement purposes in sauces [71], beverages [72], ice creams [73], low-fat [72] or bakery products [74]. Mixed with other natural compounds, it is possible to produce edible films [75]. Carob bean is used in food recipes that can benefit health [6], as antioxidants [76], because of its polyphenols and flavonoids contents; anti-diabetic effect [77], also because of its LBG, flavonoids and phenolic acids; anti-hyperlipidemia properties [78], conferred by the fibers and gastrointestinal benefits [79], thanks to the locust bean gum. It can also be used for pharmaceutical/medical purposes [80,81], in buccal [82], oral [83], gastric [84], colon [85], ocular [86], or topical [87] drug delivery [88] formulations. Recently, with the necessity of finding greener energy devices, LBG has been proposed as a component of bio-batteries [89], as the binder of ZnSO_4_ and MnO_2_, in order to form a “quasi-solid-state” LBG electrolyte. Its high specific capacity, rate performance and capacity retention, make LBG a viable ingredient with a high potential for use as a binder for green batteries.

### 2.6. Synergistic Behaviors of LBG Mixtures

Locust bean gum shows synergistic behaviors with different polysaccharides, such as xanthan gum, carrageenan and alginate, for example. As reported by Dionísio and Grenha [90], and by Verma et al. [81], the rheological synergy can be of interest for pharmaceutical applications because of the non-toxicity of the products and the different entanglement levels feasible.

**Figure 3:**
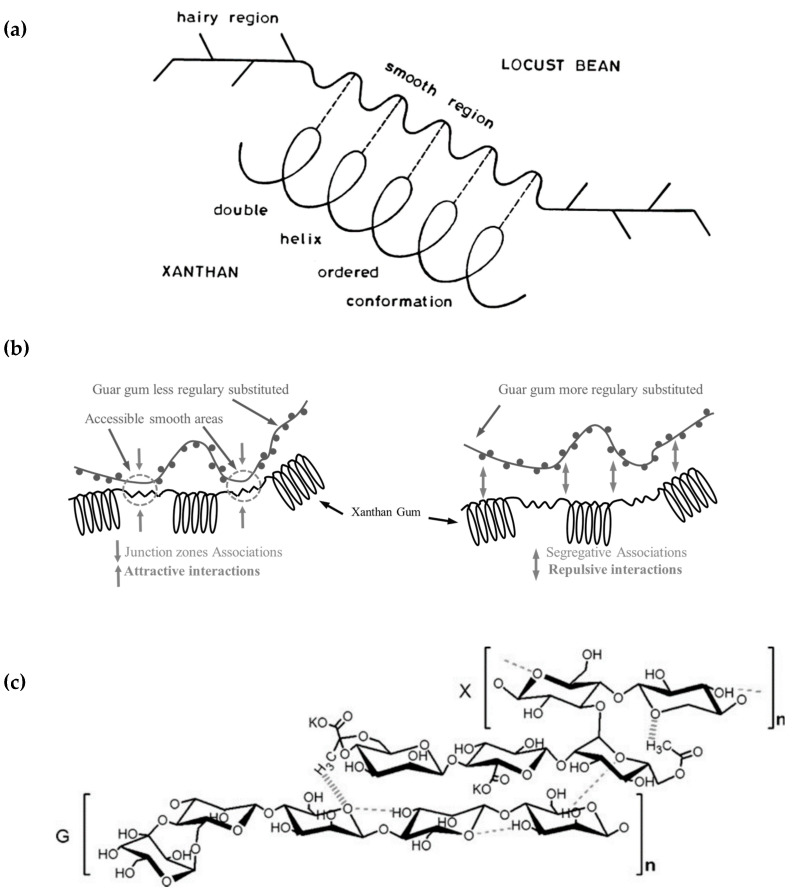
Different models of Xanthan Gum—Galactomannan synergies [98]: (**a**) Unilever model [99], (**b**) Norwich model [100], (**c**) Tako model [101] (G, galactomannan; X, xanthan gum).

The synergies between xanthan gum (XG) and galactomannans (GM) have been well studied since they were discovered. Nevertheless, the molecular mechanisms of such synergies continue to be debated. Historically, the synergistic behavior was first explained by poor or inexistent interactions because of gum incompatibility [91], volume exclusion [92], or because of weak connections other than specific intermolecular interactions [93]. Another explanation of such synergy is the existence of cooperative interactions between both polysaccharides [94]. The galactomannan branches are not regularly placed along the backbone: some parts are more branched than other sections, which are considered ‘smooth’ regions [19]. Different authors tried to explain where these interactions take place: between side chains of xanthan helices and smooth regions of the galactomannan backbones [95], between the xanthan helix and those smooth regions [96], or between the disordered xanthan and galactomannan structures [97]. Respectively, those possible scenarios are called the “Tako model”, the “Unilever model” and the “Norwich model”, as described by Takemasa and Nishinari (Figure 3) [98].

This synergistic interaction is responsible for the modifications found in the rheological properties of the solutions, such as the viscosity, depending on the XG:GM ratio [102], pH, or the GM fine structure [103,104] and on the temperature [105]. Schreiber et al. showed, by atomic force microscopy measurements, the synergies between XG and different types of GM, and explained this phenomenon by the length and the flexibility of polysaccharide chains. A separation of phase between XG and GM occurs after 2 days when both are mixed at room temperature, and a change of the mechanical properties takes place after two weeks. The addition of salt reduces the synergism by protecting the anion charge of xanthan and by lowering the gelation strength [106]. These viscosity effects are useful in the food industry [107] for the improvement of sauces formulations, for example [108,109].

Kurt and al. studied the interactions between these two polysaccharides and glycerol in order to create a biodegradable edible film [110]. The optimization of the film formulation shows a nonlinear behavior for the mechanical properties, meaning that LBG:XG:glycerol mixtures possess some synergism. Films were successfully created and potentially used for this purpose. By mixing LBG, XG and potato starch, Yu et al. improved the final product made by 3D printing, as they observed differences by changing the proportions of LBG:XG. They reported that XG improves the printing performance and gel fineness but shows more printing deviations and low shape retention ability. LBG, on the other hand, produces better mechanical properties and printing accuracy but a lower fluidity and a bad quality of the final product [111].

The LBG:XG mixtures are also applicable for drug delivery, as Sharma et al. considered [112]. The modification of LBG:XG proportions in their experimental design allowed them to prepare microparticles with more favorable delivery kinetics for celecoxib. The encapsulation of tea polyphenols has been studied by Tian et al. [113]. The polymeric beads were made by using a w/o emulsion and the tea polyphenol release was tested in PBS solutions. A sustained release was obtained, and good stability of the LBG:XG matrix was assessed without the use of synthetic emulsifiers, which is a useful innovation in the field of new delivery materials. Bektas et al. showed also the feasibility of using these mixtures in tissue engineering [114]. In that investigation, they added mastic gum to prepare cryogels. The mechanical properties, the porosity and the cytocompatibility of the matrices formed, make it useful as a bioactive agent delivery system or as scaffolds for cartilages, for example.

As mentioned above, the use of locust bean gum is viable as a binder in green batteries. Yang et al. decided to study the effect of the LBG:XG mixture and reported its effectiveness and low-cost production [115].

**Figure 4:**
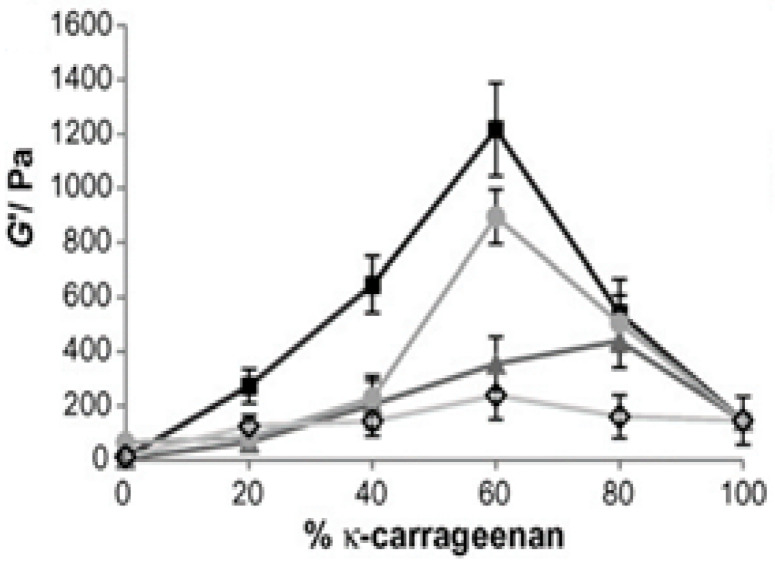
G’ elastic component of galactomannan/κ-carrageenan mixed gels (guar gum (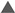); locust bean gum (■); *Gleditsia triacanthos* galactomannan (♢) and *Sophora japonica* galactomannan (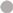)) [117].

Carrageenan and galactomannans show, in general, synergistic effects also. Turquois et al. showed the synergies between LBG and κ-carrageenan. They demonstrated the influence of the polysaccharide solution concentrations, the κ-carrageenan:LBG ratio and the influence of molecular weight [116]. Rheological tests of galactomannans/κ-carrageenan mixtures have also been performed by Pinheiro et al. (Figure 4) [117]. They demonstrated the effect of the M:G ratio and the polysaccharide microstructure on viscosity. Some previous works with ^13^C NMR showed the interactions between κ-carrageenan and LBG, which are influenced by the distribution of the galactose branched units on the backbone [118]. As for the XG:GM mixtures, the smooth regions of LBG are responsible for the entanglement between both polysaccharides. For a low total concentration, the gelation is produced by a bi-continuous two-phase system [119], one being the LBG, the other constituted by droplets or a secondary phase of κ-carrageenan. Potassium chloride also influences the gelation process, producing it more easily [120]. Light scattering [121] and small angle X-ray scattering [122] put in evidence the entanglements of carrageenan with itself and explain the role played by locust bean gum on the interaction with the charged polymer. Additionally, the extent of the double helix conformation of carrageenan, the M:G ratio and the distribution of galactose units affect the synergism. This particular synergism is useful for medical purposes such as wound healing and tissue-repairing devices [123]. As studied by Mendes de Moraes et al., the mixture κ-carrageenan:LBG permits the transdermal delivery of hydrophilic compounds [124]. The release of arbutin by this hydrogel is better than that from a commercial cream, also permitting improved skin hydration and the reduction in the melanin index while being non-toxic. The preparation of 3D printable food is feasible by combining κ-carrageenan:LBG:XG, which has resulted as particularly useful for dysphagic patients [125]. Adding alginate and through a w_1_/o/w_2_ double emulsion, Wang et al. succeeded in creating sustained and controlled delivery devices [126]. The formulation κC:LBG:chitosan:PVA, obtained by mixing all the components after dissolving each one individually, was studied by Yong et al. to prepare intelligent packaging films [127]. The product was efficient for the immobilization of anthocyanins, allowing the film to be pH and ammonia sensitive.

Although polyvinyl alcohol (PVA) is a synthetic polymer, PVA:LBG is also a well-studied combination [128]. Double-layer films made with LBG:PVA and agar:PVA to be used as shrimp freshness indicators were studied by Yao et al. [129]. They integrated red pitaya betacyanins in the LBG layer to provide a “sensitive layer” and TiO_2_ in the agar section as the “protective layer”. The film was prepared by placing an LBG:PVA solution onto the agar:PVA layer. A good light and water vapor barrier capacity have been demonstrated, as the sensitive layer worked well when exposed to different atmospheres, and TiO_2_ prevented the color changing, permitting a better color contrast when used with shrimp. The combination of LBG:carboxymethyl chitosan has also been studied by Yu et al. in order to prepare films [130]. The incorporation of natural essential oil in the structure permits a better elongation at break, a higher water resistance, and oxygen barrier properties, consequently increasing the antioxidant and antibacterial activities and improving its hydrophobicity but decreasing its water vapor barrier capacity and tensile strength. Chitosan can also be incorporated into LBG as nanoparticles to produce another type of biobased film [131]. The final product possesses a great resistance but it diminished when a natural deep eutectic solvent plasticizer was added. The combination LBG:PVA has been used with extracts of *Loropetalum chinense* var. *rubrum* petals to form another type of smart packaging [132]. Yun et al. demonstrated the antioxidant and antimicrobial activity, pH and ammonia sensitivity and its capability as a good freshness indicator. By adding betacyanins into film production, smart packagings are also feasible [133].

Locust bean gum and alginate form an interpenetrated complex, useful for drug delivery purposes because of their swelling behavior and drug release control [134,135,136,137]. The matrix is usually prepared by the ionotropic-gelation technique, using the coacervation of alginate with a divalent cation as Ca^2+^, which yields edible beads from natural raw materials. Aclofenac [135,137], capecitabine [134] and captopril [136] releases were analyzed in these types of systems. The drug delivery study showed a controlled release resulting from these matrices. This technique can also be employed for the encapsulation of tea polyphenols [138]. The preparation of edible packaging is feasible simply by mixing LBG with sodium alginate, integrating daphnetin [139] and using a CO_2_ atmosphere [140]. A low bacteria development was observed and a tasteful product was obtained.

Another polysaccharide that can be found mixed with LBG is inulin. This carbohydrate is mainly used in drug delivery, but it is valid for food applications, as studied by Góral et al. [141]. They found that the concentration of polysaccharides will decrease the cryoscopic temperature, the melting time of coconut milk-based ice cream and its hardness. A higher inulin ratio over LBG produced a higher overrun and tasteful ice cream. In the food industry, it is possible to use carboxymethyl cellulose combined with LBG to stabilize unfizzy doogh [142]. Numerous patents between locust bean gum, varied polysaccharides and proteins have been published, mostly for developing sauces or other food recipes. Table 1 summarizes the examples of the mixtures between LBG and other polymers collected in this review.

It is also feasible to prepare aerogels based on LBG and graphene oxide for water purification applications [143]. Their 3D structure has allowed the sorption of rhodamine-B and successfully remove this dye over indigo carmine thanks to the dye charge.

## 3. LBG Derivatives

### 3.1. Modifications of Functional Groups

As specified by Barreto Santos et al. and by Yadav and Maiti, galactomannan polysaccharides can easily be chemically derivatized by functions such as sulfation, carboxylation, or acetylation, for example [13,144]. This derivatization usually employs hazardous chemicals and solvents but produces non-toxic and biodegradable matrices.

**Figure 5:**
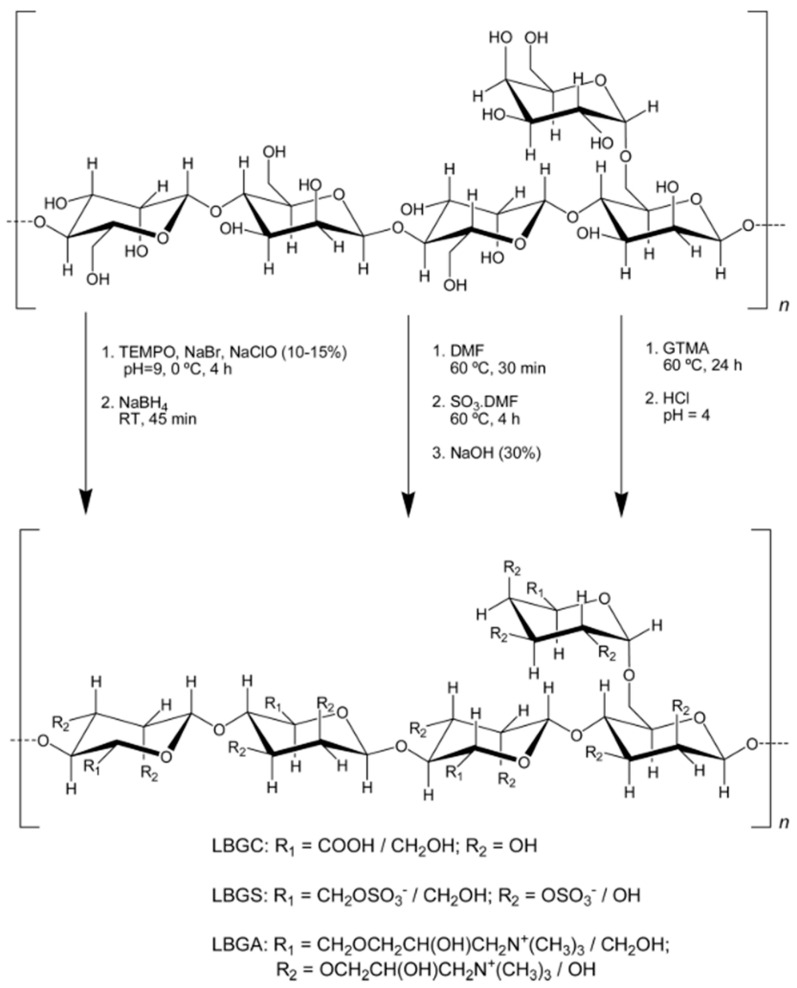
Chemical modification of locust bean gum: LBG carboxylate, LBG sulfate, LBG trimethylammonium [145].

Braz et al. decided to prepare different types of modified LBG (Figure 5) [145]. The sulfation of LBG can be performed with SO_3_DMF as reported by Braz et al. [146]. The resulting modified polysaccharide can be mixed with chitosan to form solid and compact spherical beads as a promising antigen delivery material. The carboxylation of LBG is also feasible, by using TEMPO and NaBr after an organic reaction. The last described is the grafting of a quaternary ammonium salt thanks to GTMA and HCl. The different LBG materials formed were complexed with a reverse-charged polysaccharide: for carboxylic LBG and sulfated LBG, chitosan was used. Ammonium LBG was complexed with sulfated LBG. The main purpose is to use these complexes for drug delivery applications; their toxicological evaluation shows that the ammonium derivative presents severe cytotoxicity but it reverted when complexed with sulfated LBG.

The carboxymethylation of LBG is possible by using monochloroacetic acid to generate an efficient drug delivery matrix [147]. The synthesis permits a good degree of substitution but with a decrease in the viscosity and the molar mass. As reported by Katy et al., the CMLBG is “safe enough for internal use” and recommends the usage of CMLBG:PVA interpenetrated network microbeads crosslinked with glutaraldehyde for controlled oral drug delivery. A greener way to employ CMLBG is to combine it with alginate and use Al^3+^ as a crosslinker [148]. The resulting IPN features depend on the gelation time, the higher, the better the release behavior. Al^3+^ is also an ionic crosslinker for CMLBG and CMC by single water-in-water emulsion gelation processes with applicability for drug delivery [149]. Glipizide [148,150] and diclofenac [149] releases have been studied using these networks.

LBG can also be derivatized with inorganic components, such as palladium to transform it into a green catalyst [151]. The Pd insertion is made thanks to Pd(OAc)_2_ reacting with LBG in water and through ultrasonic irradiation at 80 °C. The precipitate after cooling, recovered by adding ethanol, is then filtrated and isolated. Following this procedure, the Pd is inserted on the polysaccharide-reduced ends. Ben Romdhane et al. tried different reactions using Pd@LBG as the catalyst and succeeded in recuperating a good yield and regenerating the catalyst five times. Another example has been prepared by Tagad et al.: an LBG derivatized by gold nanoparticles [152]. The HAuCl_4_ is introduced into a solution of LBG and autoclaved at 120 °C and 15 psi. 4-nitrophenol to 4-aminophenol reductions were efficiently catalyzed by Au@LBG. When doped with SnO_2_, it shows a fast response and good ethanol-sensing behavior.

Singh et al. have successfully grafted polyacrylamide functions onto LBG by microwave-initiated graft copolymerization techniques [153]. They proved its non-morbidity and toxicity in different organs and a controlled release of budesonide in the colon. Jin et al. grafted methyl acrylate and acrylic acid from LBG by Fenton reactions [154]. The grafting affects the viscosity, contact angle, water solubility and mechanical properties. Adhesion to polyester fiber makes it useful for textile applications. Another polymer family grafted from crosslinked LBG by means of divinyl sulfate are polyethyleneimines [155]. Good blood compatibility, an easily modifiable polysaccharide and a good, controlled release behavior made it a promising drug carrier.

Finally, the network structure constituted by sodium acrylate:LBG:N,N’-methylenebisacrylamide has been prepared by irradiation in order to create a superabsorbent polymer [156].

### 3.2. Crosslinking Reactions

It has been reported that glutaraldehyde is a potential crosslinker for several polysaccharides with chitosan [157]. Jana et al. showed the possibility to form LBG:CS matrices for drug delivery. It suppressed the burst release allowing a sustained release [158]. In addition, glutaraldehyde can crosslink a single LBG to produce drug delivery matrices [159].

Citric acid, used as an LBG crosslinker through solventless reactions with basic catalysts, has been studied by Petitjean et al. [160]. Provided that a temperature above 170 °C and a sufficient reaction time (>20 min) are used, the crosslinking process was produced with a good yield, resulting in a remarkable swelling behavior and dye sorption capabilities. By functionalization of these LBG networks with β-cyclodextrin, specific interactions between some sorbates and LBG are enhanced [161]. Besides, by adding lignin to the initial mixtures, the amounts of polyphenolic compounds sorbed were also significantly increased [162]. Another crosslinking process has been reported by Hadinugroho et al. [163]. LBG was swollen before being UV-cured with citric acid and an acidic catalyst. Then, the resulting product was washed with acetone and dried at ambient temperature. They showed that, under acidic conditions, the protonation of the C6 hydroxyl group of mannose and galactose was easier, and so they concluded that the crosslinking was taking place at this carbon site [163,164]. The disintegration of the tablets produced by these materials has been also studied by this group [165].

## 4. Conclusions and Perspectives

Among the galactomannans, locust bean gum has a mannose-to-galactose ratio of about 4:1 and a minimally branched structure and it needs heat to fully hydrate. Although it does not gel on its own, LBG forms gels with other hydrocolloids. LBG is non-digestible and may be classified as a soluble fiber. An efficient stabilizer in the food industry, its water-binding and thickening properties promote also its uses to improve the gel properties of some hydrocolloids.

In addition to those well-known applications in the food sector, the feasibility of producing several types of ‘green’ matrices using LBG, either by itself or derivatized or combined, has also been explored recently. Those materials, produced either by physical entanglements or chemical crosslinking reactions, can be used in other fields, including packaging, biopharmaceutical devices, batteries, catalysts, etc.

As for the hydrocolloid market, LBG prices were similar in 2020 to those of alginates, pectin or agar (ca. 18 $/kg) [9]. Nevertheless, the excellent properties of LBG for some applications in the food industry and its relatively low production volume (well below that of guar gum, for instance) have produced a shortage in the market. Carob trees take over a decade to become productive, so an increasing demand cannot be met simply by planting more trees.

Other alternatives start being used as LBG replacements, and these gum hydrocolloids are often employed in combinations. The question is whether those replacements are satisfactory enough to meet the food and beverage industry needs, so a higher-priced LBG can be used for more “selective” purposes in other fields. In this review, two interesting characteristics of locust bean gum have been highlighted. On the one hand, its synergisms with other biobased polymers are remarkable, widening its range of potential applications. On the other hand, the possibilities of derivatizing its chains and/or creating crosslinking bridges are also of great interest in order to explore other possibilities.

## Figures and Tables

**Table 1:** Examples of synergies between LBG and other biobased polymers, method of preparation of the mixtures and their uses.

Biobased Polymer Coupled	Preparation Method	Use	Reference
XG	Mixture	Food industry	[107,108,109]
XG, Glycerol	Mixture	Edible film	[110]
XG, potato starch	Mixture	3D printing	[111]
XG	Emulsion w/o	Drug delivery	[112]
XG	Emulsion w/o	Encapsulation	[113]
XG, mastic gum	Freeze dried from Mix	Tissue engineering	[114]
XG	Mixture	Binder in green battery	[115]
ι-, κ-Carrageenan, Gelatin	Mixture	Wound healing, Tissue repairing	[123]
κ-Carrageenan	Mixture	Transdermal delivery	[124]
κ-Carrageenan, XG	Mixture	Food 3D printing	[125]
κ-Carrageenan, alginate	Double emulsion w_1_/o/w_2_	Delivery device	[126]
κ-C: Chitosan: PVA	Mixture	Film packaging	[127]
PVA + agar: PVA	Mixture and double layer	Film packaging	[129]
Carboxymethyl Chitosan	Mixture	Film packaging	[130]
Chitosan	Mixture	Biobased films	[131]
PVA	Mixture	Smart packaging	[132,133]
Alginate	Mixture + ionic gelation	Drug delivery	[134,135,136,137]
Alginate	Mixture + ionic gelation	Encapsulation	[138]
Alginate	Mixture	Edible packaging	[139,140]
Carboxymethyl Cellulose	Mixture	Food	[142]
